# Supplementary material for: Perinatal mental health in India in the states of Haryana and Telangana: A district-level situational analysis
Source: Glob Ment Health (Camb). 2025 Jun 30;12:e93. doi: 10.1017/gmh.2025.10021 (PMC12394031; doi:10.1017/gmh.2025.10021)
Supplement: Mellers et al. supplementary material [file S2054425125100216sup001.docx]

Supplementary Figure S1: Map displaying location of Haryana and Telangana


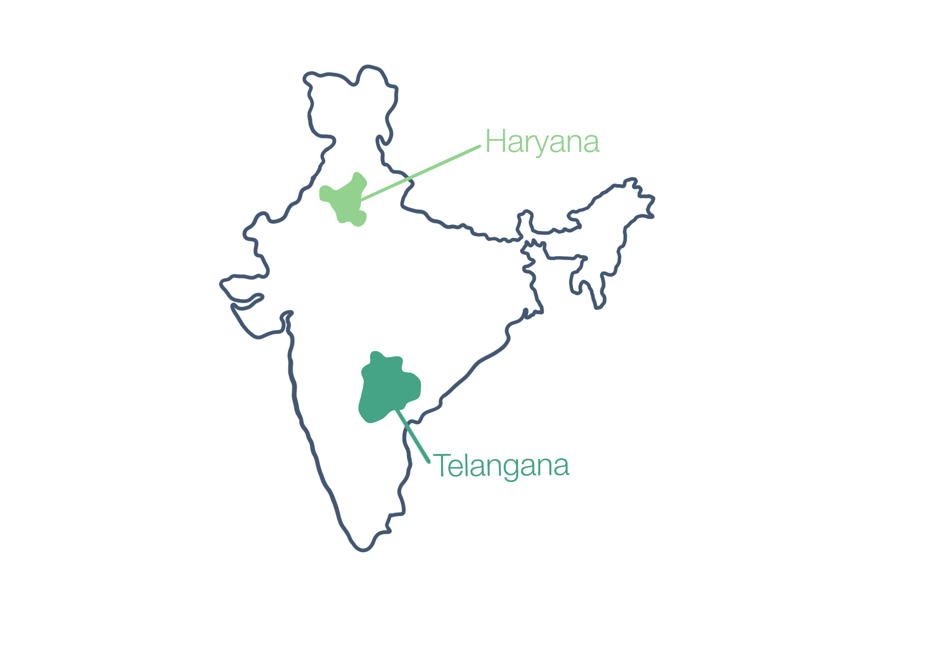


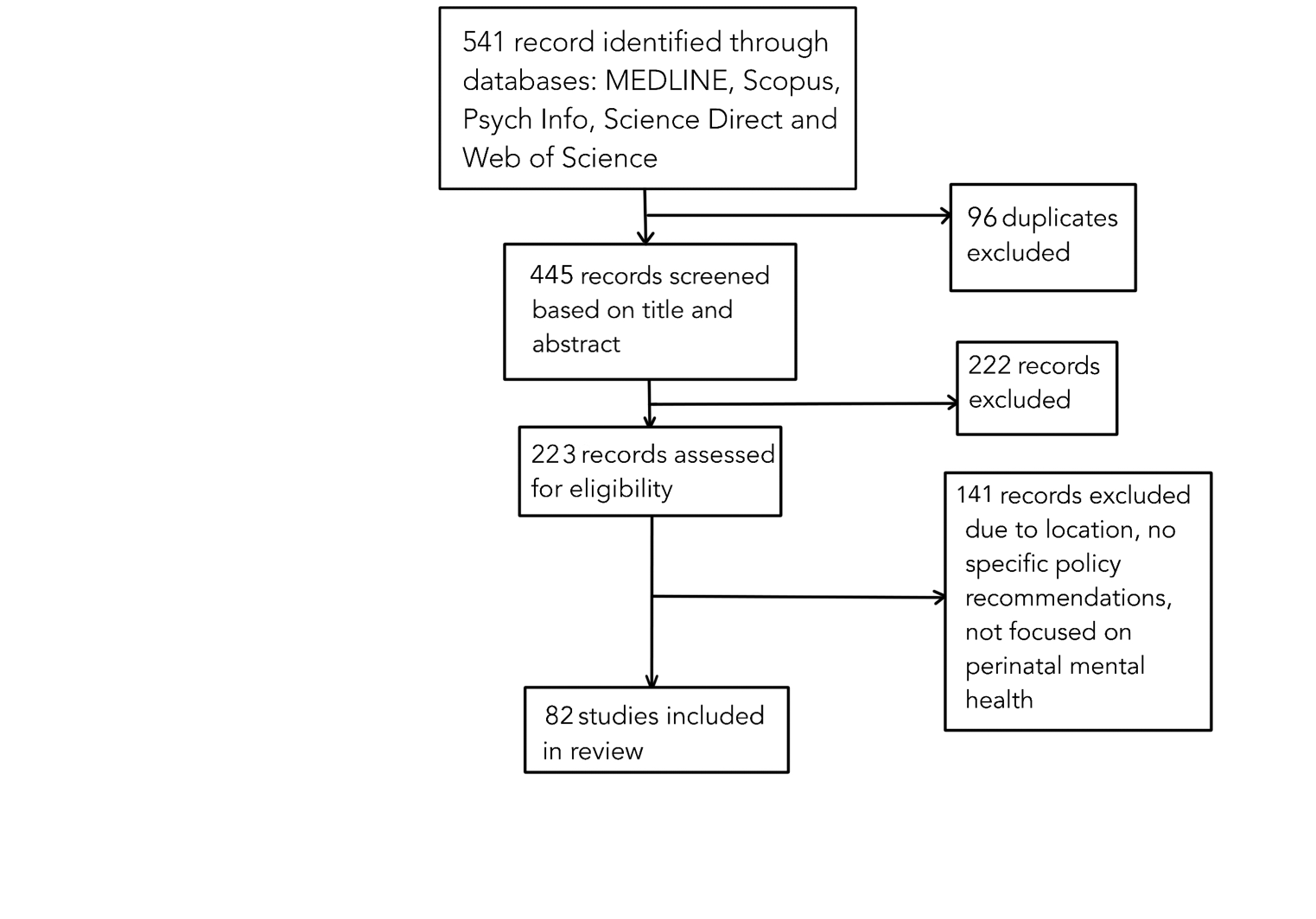
Supplementary Figure S2: Article Screening Process


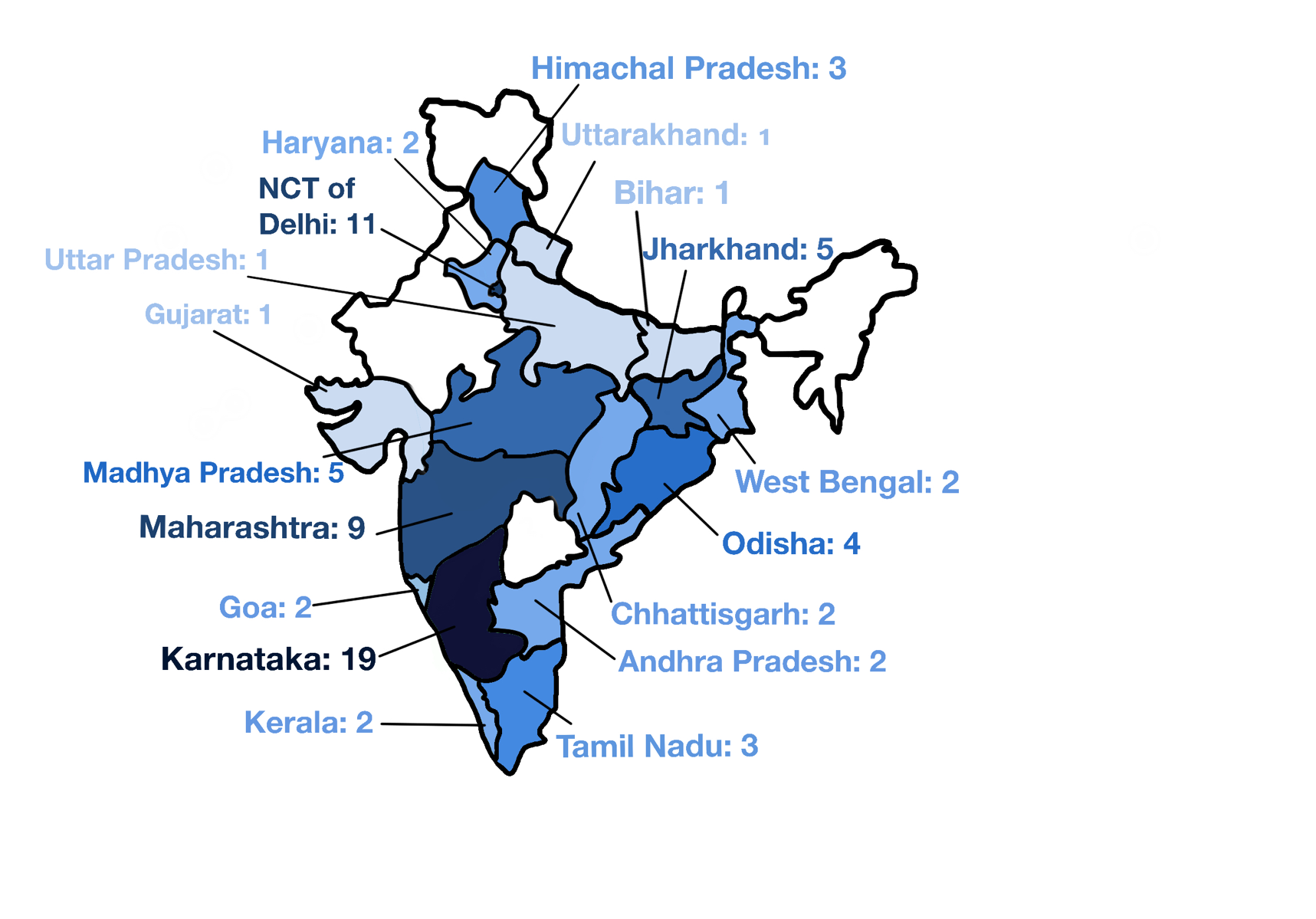
Supplementary Figure S3: Study Location Map

*Search Strategy for Pub Med search*

((Antenatal depression[MeSH Terms]) OR (postnatal depression[MeSH Terms]) OR (Maternal mental health)) OR ((maternal[Title/Abstract]) AND (mental illness[Title/Abstract])) OR ((perinatal[Title/Abstract]) AND (mental health [Title/Abstract])) AND ((Policy) OR (Plan*) OR (Polit*) OR (Health systems) OR (legislation) OR (maternal health services[MeSH Terms])) AND ((India) OR (Telangana) OR (Haryana))

*Search Strategy for Scopus*

( TITLE-ABS-KEY ( "Perinatal mental health" OR "maternal mental illness" OR "antenatal depression" OR "postnatal depression" ) AND TITLE-ABS-KEY ( "India" OR "Telangana" OR "Haryana" ) AND ALL ( "Policy" OR "Plan*" OR "Health system" OR "Polit*" OR "mental health service" ) )

*Search Strategy for Web of Science*

((TS=("antenatal depression" OR "postnatal depression" OR "maternal mental health"OR "maternal mental illness" OR "perinatal mental health" )) AND ALL=("Policy" OR Plan* OR Polit* OR "Health systems" )) AND ALL=("India" OR "Haryana" OR "Telangana" )

OR

((TI=(“mental disorders” AND “pregnant”)) AND ALL=(“India” OR “Haryana” OR “Telangana”))

*Search Strategy for Psych Info*

TITLE("perinatal mental health" OR "maternal mental illness" OR "antenatal depression" OR "postnatal depression" ) AND IF("India") OR IF( "Telangana") OR IF("Haryana" ) AND FULLTEXT("health systems") OR FULLTEXT("policy") OR FULLTEXT("plan" )

*Search Strategy for Science Direct*

Title/Abstract/keywords: perinatal mental health OR postpartum depression OR maternal mental illness OR antenatal depression

AND

All terms: India AND policy AND system

*Exclusion criteria*

1) Not focused on perinatal mental health conditions, 2) No policy/health system/legislation recommendation, 3) Only mention of policy is generic without specific suggestion.

Supplementary Materials Table S1: Indices of Maternal Health

| MATERNAL HEALTH SERVICES | National level - India | | State level-Haryana | | State level - Telangana | |
| --- | --- | --- | --- | --- | --- | --- |
| Antenatal Clinic attendance | At least once | Four times | At least once | Four times | At least once | Four times |
|  | 82.7% | 58.1% | 82.3% | 60.4% | 96.6% | 70.4% |
| HIV testing | Offered | Results not received | Offered | Results not received | Offered | Results not received |
|  | 81.6% | 54.1% | 76% | 55.2% | 88% | 24.4% |
| Adequate tetanus toxoid protection received | Overall | Rural | Overall | Rural | Overall | Rural |
|  | 92% | 91.7% | 90.7% | 91.7% | 89.6% | 89.7% |
| Attendance of postnatal care within 2 days of delivery | Overall | Rural | Overall | Rural | Overall | Rural |
|  | 78% | 75.4% | 91.3% | 90.8% | 87.6% | 97.3% |

Supplementary Materials Table S2: Indices of Maternal Health (district level)

| MATERNAL HEALTH SERVICES | District level - Faridabad | District level - Siddipet |
| --- | --- | --- |
| Antenatal Clinic attendance four times | 51.2% | 62.6% |
| Adequate tetanus toxoid protection received | 81.4% | 89.8% |
| Attendance of postnatal care within 2 days of delivery | 93.3% | 74.1% |

Supplementary Materials Table S3:Search results for perinatal mental health within mental, maternal and child health programmes

| Policy/Guideline/strategy | Mention of specific perinatal mental health focus | Any relevant points |
| --- | --- | --- |
| National mental health policy  2014 | No | Increase access to services for vulnerable groups including women and children.  Highlights the importance of the mother and child bond and suggests that auxiliary nursing midwives should have the opportunity for up-skilling in mental illness |
| National mental health program  1982 | Not that can be ascertained from National Health Mission or Directorate general of Health services website |  |
| District mental health program  1996 | Not that can be ascertained from various reviews^1,2^ | Provide mental health services at the community level and integrate them into primary health care services |
| Mental Healthcare Act  2017 | No | Mandates joint infant mother care if mother admitted for management of mental health condition |
| Reproductive, Maternal, Newborn, Child and Adolescent Health (RMNCH+A) strategy  2013 | No | Highlights importance of tackling adolescent mental health |
| Janani Suraksha Yojana 2005 | No | Aims to reduce maternal and neonatal mortality by promoting institutional delivery – cash incentive for delivery in government heath centres or accredited private institutions. Advocates for at least 3 ANC checkups and a post natal visit within 7 days of delivery |
| Janani Shishu Suraksha Karyakram 2011 | No | Ensures free services to all pregnant women and sick neonates (up to 30 days after birth) accessing public health institutions.  All expenses related to delivery in public institution to be borne by government.  Although doesn’t specify whether this would include admission with a mental health crisis. |
| DAKSHATA (empowering providers for improved maternity and newborn health MNH care during institutional deliveries)  2015 | No | Improve the competency of providers of care during the intra and immediate postpartum period and ensure essential supplies in the labour room are in place. |
| Prashan Mantri Surakshit Matritva Abhiyan PMSMA  2016 | No | Provide fixed-day assured comprehensive and quality antenatal care universally to all pregnant women in the 2^nd^ and 3^rd^ trimester on the 9^th^ of every month.  Visits should include individual/group counselling on danger signs during pregnancy and a safe motherhood booklet can be provided. In addition, counselling programs for the management of unintended/unwanted pregnancy and contraception. No specific mention of mental illness. |
| LAQSHYA Labour room quality improvement initiative  2017 | No | Aim to provide respectful and ‘zero-defect’ care for all pregnant women and newborns. Patient satisfaction is a measured outcome but reports of birth trauma or rates of PND or anxiety are not measured.  Mentions identifying ‘vulnerable patients’ and measures taken to protect them from harm. |
| IEC guidelines | No | Postpartum presentation slides include asking about feeling unhappy or crying easily.  History taking presentation includes asking about domestic violence.  Counselling presentation details intermediate and long term affects of domestic violence on mother and child but does not mention mental health impact specifically.  Quality of care presentation covers the possible reactions of women following a complication listing depression, disorientation, denial, guilt and anger. |
| Maternal health guidelines on National Health Mission website | Guidelines for antenatal care and skilled attendance at birth include asking on post natal visit 2 and 3 ‘Does she feel unhappy or cry easily? This indicates post-partum depression and usually occurs 4-7 days after delivery. Assure her that everything will be fine and refer her to the MO only if the problem persists’.  Guidelines of maternal health services during COVID pandemic: mentions evidence related to increased rates of maternal depression and also lists postpartum mood changes as a complication to be assessed.  Guidelines for the Operationalisation of Midwifery Units: in list of conditions postnatal mental health conditions should be identified and managed collaboratively/referred on. | Safe motherhood book: does not mention mental illness or warning signs, but emphasises the importance of emotional support from family members during pregnancy and child birth. |
| Federation of Obstetrics and Gynaecological Societies of India (FOGSI) website and annual reports | No guideline published for the management of perinatal mental illness | FOGSI made maternal mental health their national priority in 2019.  In 2016/17 a public campaign where free TVs displaying health talks on various subjects including postpartum depression was introduced in FOGSI members’ waiting rooms (MAMTA TV).  Safe motherhood committee that covers aspects of postnatal care including mental health. |
| National tele mental health programme in India  (Course Content for Tele MANAS Counsellors)  (Point of Care Guide for Tele MANAS Counsellors) | Yes – Module 12 is focused on mental health issues in Women and Handling Issues Related to Gender Based Violence. Includes perinatal mental illness.  Point of care guide includes treatment/referral pathway for symptoms of perinatal mental illness. | Aims to provide free tele mental health support to people 24/7 |
| National Suicide Prevention strategy | No | Objectives include establishing effective surveillance mechanisms for suicide, psychiatric OPD services with suicide prevention services through the DMHP and integrate mental well-being into the curriculum. Also reduce access to suicide modes and leverage media to create awareness and de-stigmatise mental illness.  Mentions augmenting short-term training of non specialist doctors, psychologists, social workers, nurses, and community health workers under relevant mental health programmes (but maternal health programmes not explicitly mentioned).  ‘Strengthen suicide prevention efforts targeting women’ including education, providing economic security, empowering women and reducing violence against women, but no specific mention of targeting women in perinatal period.  Provide suicide prevention to family members. |
| Ayushman Bharat (2018) | No | Health and Wellness centres to be created to deliver primary health care with a focus on wellness and the delivery of a wide range of services including mental healthcare services.  Pradhan Mantri Jan Arogya Yolanda is a health assurance scheme for coverage of mental disorders amongst other illnesses. This scheme includes mood disorders, delusional disorders, stress related disorders, behavioural disorders and substance abuse related disorders. Also details wide range of treatment and investigations that would be covered. |

Kirpekar, Vivek; Faye, Abhijeet1; Bhave, Sudhir; Gawande, Sushil; Tadke, Rahul. District mental health program: Then and now. Indian Journal of Psychiatry 66(7):p 603-613, July 2024. | DOI: 10.4103/indianjpsychiatry.indianjpsychiatry_974_23

2 Amiti Varmar; Karen Mathias; Thara Rangaswamy. Deconstructing the DMHP: A critique of the District Mental Health Programme. India Mental Health Observatory, June 2021. https://cmhlp.org/wp-content/uploads/2021/08/Issue-Brief-DMHP-IV.pdf
